# Basal and Adrenocorticotropic Hormone Stimulated Plasma Cortisol Levels Among Egyptian Autistic Children: Relation to Disease Severity

**DOI:** 10.1186/1824-7288-36-71

**Published:** 2010-10-30

**Authors:** Rasha T Hamza, Doaa H Hewedi, Mona A Ismail

**Affiliations:** 1 Department of Pediatrics, Faculty of Medicine, Ain Shams University, Cairo, Egypt; 2 Department of Psychiatry, Faculty of Medicine, Ain Shams University, Cairo, Egypt; 3 Department of Clinical Pathology, Faculty of Medicine, Ain Shams University, Cairo, Egypt

## Abstract

**Background:**

Autism is a disorder of early childhood characterized by social impairment, communication abnormalities and stereotyped behaviors. The hypothalamic-pituitary-adrenocortical (HPA) axis deserves special attention, since it is the basis for emotions and social interactions that are affected in autism.

**Aim:**

To assess basal and stimulated plasma cortisol, and adrenocorticotropic hormone (ACTH) levels in autistic children and their relationship to disease characteristics.

**Methods:**

Fifty autistic children were studied in comparison to 50 healthy age-, sex- and pubertal stage- matched children. All subjects were subjected to clinical evaluation and measurement of plasma cortisol (basal and stimulated) and ACTH. In addition, electroencephalography (EEG) and intelligence quotient (IQ) assessment were done for all autistic children.

**Results:**

Sixteen% of autistic patients had high ACTH, 10% had low basal cortisol and 10% did not show adequate cortisol response to ACTH stimulation. Autistic patients had lower basal (p = 0.032) and stimulated cortisol (p = 0.04) and higher ACTH (p = 0.01) than controls. Childhood Autism Rating Scale (CARS) score correlated positively with ACTH (r = 0.71, p = 0.02) and negatively with each of basal (r = -0.64, p = 0.04) and stimulated cortisol (r = -0.88, p < 0.001). Hormonal profile did not differ in relation to EEG abnormalities, IQ and self- aggressive symptoms.

**Conclusions:**

The observed hormonal changes may be due to a dysfunction in the HPA axis in autistic individuals. Further studies are warranted regarding the role of HPA axis dysfunction in the pathogenesis of autism.

## Introduction

Despite the fact that autism was described more than 60 years ago, its etiology remains a mystery [1-5]. Many genetic studies [6-8], hormone and neurotransmitter analysis [9-15], did not find a reasonable explanation for the mechanisms underlying development of autism. Lesions of different brain regions, including the limbic system, have been implicated in the development of autism [12,16]. The HPA axis deserves special attention, since it is the basis for emotions and social interactions, that are affected in autism [12,13,15,17].

One of the approaches to test the HPA axis is a measurement of its hormones and their subsequent targets together with the rapid ACTH stimulation test [18,19]. Studies regarding abnormalities in the HPA axis in autistic patients showed conflicting results [20-22].

With this background, this study was conducted to assess plasma cortisol (morning basal and ACTH stimulated), and ACTH levels in autistic children and adolescents and their relationship to disease characteristics in terms of disease severity, IQ, EEG abnormalities and self-aggressive behaviour.

## Subjects and Methods

This cross sectional case-control study was conducted on 50 autistic children and adolescents diagnosed according to the 4^th ^edition of Diagnostic and Statistical Manual of Mental Disorders (DSM IV) [23]. Patients were recruited from the Institute of Psychiatry (n = 36) and Pediatric Psychiatry Clinic, Children's hospital (n = 14), Faculty of Medicine, Ain Shams University, Cairo, Egypt during the period from the beginning of May 2009 to the end of November 2009. They were 40 males and 10 females with a male: female ratio of 4:1. Their ages ranged between 3 and 12 years with a mean age of 7.35 ± 2.6 years. None of the patients had underlying conditions apart from autism (syndromic causes, chromosomal or metabolic abnormalities) that could influence HPA axis function independently of autism.

Autistic patients were studied in comparison to 50 healthy age-, sex- and pubertal stage- matched children and adolescents serving as controls. The latter had no clinical findings suggesting neuropsychiatric manifestations nor endocrine abnormalities in particular adrenocortical function (normal blood pressure, normal serum sodium, potassium and random blood sugar). None of the studied subjects were taking medications that might influence the HPA axis.

An informed written consent of participation in the study was signed by the parents or legal guardians of the studied subjects. This study was approved by the Bioethical Research Committee, Faculty of Medicine, Ain Shams University hospitals, Cairo, Egypt.

### All studied children were subjected to

**1) Medical history **taken from the patients' caregivers laying stress on developmental history and history suggestive of adrenocortical dysfunction e.g weakness, fatigue, anorexia, nausea, vomiting.

**2) Physical examination **especially hyperpigmentation of skin and/or mucous membranes (due to high ACTH) and Tanner staging for assessment of pubertal status according to the standards of Tanner and Whitehouse [24].

3) Neuropsychiatric assessment (for patients only):

▪ Diagnosis of autism using DSM IV criteria [23].

▪ The severity of autism was evaluated using Childhood Autism Rating Scale "CARS" [25] which rates the child on a scale from one to four in each of 15 areas (relating to people; emotional response; imitation; body use; object use; listening response; fear or nervousness; verbal communication; non-verbal communication; activity level; level and consistency of intellectual response; adaptation to change; visual response; taste, smell and touch response and general impressions). According to the scale, scores of 30-36 indicate mild to moderate autism and scores of 37-60 indicate severe autism.

▪ **IQ assessment using Wechsler Intelligence Scale for Children (WISC): **[26]

Cognitive function (memory, attention, language, concept formation, problem solving, executive and visuospatial functions) was assessed with age-appropriate, translated and validated psychometric instruments that were administered by a well-trained psychologist. This scale is the most commonly used test to assess cognitive function in children. It measures verbal IQ (information and general knowledge, comprehension, arithmetic abilities, similarities to measure abstraction, vocabulary and digit span), performance IQ (picture completion for visuoperceptive defects, block design to measure left-right dominance, picture arrangement to measure subject's cognitive style, object assembly to measure organization and digit symbol) and full scale IQ. Subnormal intellectual function is diagnosed when IQ is below 70.

▪ History of self-aggressive behaviour including pulling their hair, ears and scratching their skin.

**4) Measurement of regional brain electrical activity of autistic children**. This was done by using inter-ictal EEG (for patients only).

5) Laboratory investigations:

▪ Fasting morning plasma cortisol (9:00 am)

▪ Fasting morning plasma ACTH (9:00 am)

Plasma cortisol and ACTH were assayed by a single person blind to the clinical conditions of the patients using Siemens cortisol and ACTH kits with the Immulite 2000 Analyzer using chemiluminescent immunometric assay [27]. Reference age- and sex- matched values for basal plasma cortisol ranged between 7-25 ug/dl while those for plasma ACTH ranged between 10-48 pg/ml.

▪ Rapid ACTH stimulation test: A synthetic human ACTH is used, fasting is not required and the test can be performed at any time of the day. A baseline cortisol sample is obtained and ACTH is administered in a dose of 0.25 mg intravenously and another sample for plasma cortisol is obtained at 60 minutes following the injection. Normally, the plasma cortisol response at 60 minutes post injection should reach ≥ 18 ug/dl [18].

### Statistical analysis

The results were analyzed using the Statistical Package for the Social Science (SPSS) version number 10, Echosoft corp; USA, 2005. Description of quantitative variables was in the form of mean ± standard deviation and range while that of qualitative variables was in the form of frequency and percentage. Chi-square test was used to compare 2 qualitative data. Student's t-test of 2 independent samples was used to compare 2 quantitative variables while paired t-test was used to compare plasma cortisol levels before and after ACTH stimulation. Pearson correlation coefficient test (r-test) was used to rank different variables against each other either directly or indirectly. A p value of < 0.05 was considered significant.

## Results

Of 50 studied patients, 29 (58%) were pre-pubertal, 13 (26%) were in Tanner stage 2, 7 (14%) were in Tanner stage 3 and 1 (2%) was in Tanner stage 4 which is earlier than what is normally expected for age because similar to the known combination of central nervous system disorders and precocious puberty, patients with autism may be susceptible to early onset of puberty [28]. Regarding autistic severity measured by CARS; 26 patients (52%) were of mild to moderate severity (18 males and 8 females) and 24 (48%) were severely autistic (22 males and 2 females).

None of our patients had history suggestive of adrenocortical dysfunction nor hyperpigmentation of the skin and/or mucous membranes. The mean values of all hormonal parameters among the whole sample were within normal reference ranges [Table 1]. Eight patients (16%) had a higher plasma ACTH than age- and sex- matched reference ranges, 5/50 (10%) had a lower plasma cortisol than age- and sex- matched reference ranges and 5/50 (10%) did not show adequate cortisol response to ACTH stimulation, that is, a cortisol level of < 18 ug/dl 60 minutes after ACTH injection. Autistic children had lower morning basal cortisol (p = 0.032), higher ACTH (p = 0.01) and lower cortisol response after ACTH stimulation (p = 0.04) when compared to healthy age-, sex- and pubertal stage- matched controls [Table 1].

**Table 1:** Comparison of basal and ACTH stimulated plasma cortisol, and ACTH levels among autistic patients and controls.

	Patients	Controls	t	p
Morning basal cortisol (ug/dl)	11.02 ± 5.23 (2.41-19.29)	18.94 ± 3.05 (10.32-24.10)	5.67	0.032*
Morning ACTH (Pg/ml)	31.89 ± 10.17 (18.82-56.24)	21.89 ± 6.15 (12.67-34.21)	6.28	0.01*
Cortisol 60 minutes after ACTH stimulation (ug/dl)	22.92 ± 3.12 (14.38-28.08)	27.01 ± 2.19 (18.23 ± 32.81)	4.39	0.04*

Results are expressed as mean± SD and range, p < 0.05*: significant, ACTH: adrenocorticotropic hormone.

**Figure 1:**
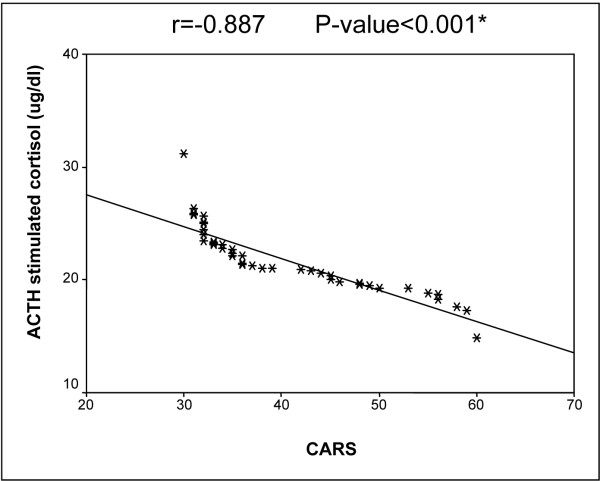
**Correlation between ACTH stimulated cortisol and autistic severity assessed by CARS**.

Moreover, significantly lower morning basal cortisol (p = 0.041), higher ACTH (p = 0.033) and lower cortisol response after ACTH stimulation (p = 0.04) were encountered as the autistic severity increased [Table 2]. In addition, CARS score correlated positively with ACTH (r = 0.71, p = 0.02) and negatively with each of basal cortisol (r = - 0.64, p = 0.04) and cortisol after ACTH stimulation (r = - 0.88, p < 0.001, Figure 1).

**Table 2:** Relationship between hormonal profile and autistic severity.

	Mild to moderate (n = 26)	Severe (n = 24)	t	p
Morning basal cortisol (ug/dl)	12.96 ± 4.28 (5.32-19.29)	8.15 ± 4.14 (2.41-16.32)	3.90	0.041*
Morning ACTH (Pg/ml)	26.94 ± 5.02 (18.82-49.61)	33.83 ± 6.08 (20.89-56.24)	4.32	0.033*
Cortisol 60 minutes after ACTH stimulation (ug/dl)	23.42 ± 4.01 (17.10-28.08)	19.94 ± 1.89 (14.38-22.92)	5.29	0.040*

Results are expressed as mean± SD and range, P < 0.05*: significant, ACTH: adrenocorticotropic hormone

Thirty three of our 50 autistic children (66%) had subnormal intellectual function (IQ below 70) among whom 25 (50%) had mild mental retardation (IQ = 50-69) and 8 (16%) had moderate mental retardation (IQ = 35-49). Of the 33 retarded patients, 20 (60.6%) had severe autism and the remaining 13 (39.4%) had mild to moderate autism. On comparing cortisol (basal and ACTH stimulated) & ACTH levels in relation to IQ of autistic patients, all hormonal parameters did not differ significantly according to the IQ of autistic children (p > 0.05).

EEG abnormalities were found in 20/50 (40%) of autistic children (14 had severe autism and 6 had mild to moderate autism). These abnormalities included focal epileptogenic activity in frontal or temporal lobes in 10 patients, generalized epileptogenic activity in 6 patients and immature background in the remaining 4 patients. None of the hormones differed between autistic children with (n = 20) and without EEG abnormalities (n = 30, p > 0.05).

Also, none of the hormones differed between autistic subjects with (n = 16) and without (n = 34) self-aggressive symptoms and among different Tanner stages (p > 0.05).

## Discussion

Autism is a disorder of early childhood characterized by social impairment, communication abnormalities and stereotyped behaviours [23]. Inspite of the fact that many studies aimed at determining the neurobiological basis of this disorder, its cause remains obscure [21].

The limbic system deserves special attention in autism because this brain region is a basis for emotions and social interactions (which are abnormal in autism). Since the limbic system influences the functions of the hypothalamus and the pituitary gland, it is logical to postulate that analyzing blood levels of pituitary hormones may reflect possible alterations of the limbic system functioning in individuals with autism [29].

In the current study, serum cortisol levels were lower and plasma ACTH levels were higher in individuals with autism, compared to normal subjects with 10% and 16% having low cortisol and high ACTH respectively. In addition, 10% did not show adequate cortisol response to ACTH stimulation. Serum cortisol and ACTH levels were analyzed in several studies but the results were inconsistent. Marinovic-Curin and co-workers [21] found lower cortisol, higher ACTH and inadequate cortisol response after ACTH stimulation among their autistic patients in comparison to controls which goes with our results. Other studies that did not perform an ACTH stimulation test did not find a difference in cortisol levels among autistic patients and controls [22,30] with only ACTH being significantly higher among cases than controls [30] or found significantly lower cortisol levels among autistic patients than controls [31]. In addition, Tani and associates [32] found lower cortisol and higher ACTH levels among patients with Asperger syndrome than controls. The difference between our findings and other studies could reflect variations in methods for hormone measurement. Also, our samples were collected in an earlier time period (9 am) and larger numbers of autistics and controls took part in our study compared to previous reports.

Cortisol has an important role in proper emotional development and functioning. Thus, abnormal cortisol levels were found in chronic depression and suicide prone behaviour [33]. In addition, lower levels of cortisol were found among holocaust victims with posttraumatic stress disorder and were pointer to "vulnerability" for its development [34]. Also, primary disorders of cortisol metabolism (Cushing syndrome and Addison disease), as well as cortisol supplement therapy are often associated with emotional disturbances [35]. Our finding of lower cortisol levels among individuals with autism is hard to interpret. Some authors hypothesized that individuals with autism display heightened response to stressors, namely venipuncture procedure so that anxiety and situational stress could explain the raised ACTH values [30] but elevated ACTH in our study was accompanied by lower cortisol levels which is in contradiction to such a conclusion. Also, the stress of venipuncture procedure was the same in both groups and so, the observed differences may be related to autism pathophysiology rather than to acute stress. There are many studies of the plasma levels of B-endorphin in autistic patients and most of them found higher levels of this hormone in individuals with autism [36-38]. Since ACTH and B-endorphin are secreted in equi-molar quantities from same precursor, pro-opiomelanocortin, from the anterior pituitary, other studies [30,36] showing elevated B-endorphin levels in autistic individuals support our finding of elevated ACTH. Finally, we hypothesize that lower cortisol and higher ACTH levels may signal a state of low basal functioning of HPA axis in autistic individuals. This was confirmed in our study by failure of 10% of autistic patients to show an adequate cortisol response to ACTH stimulation which was supported by another study [21]. Suggested mechanisms include either an abnormality in delivery of ACTH to adrenal cortex receptors, or in biosynthesis of cortisol, or in negative feedback inhibition of HPA axis by cortisol and ACTH [30-32]. This hypothesis also, sheds light on the fact that HPA axis dysfunction might have a role in the pathophysiology of autism and its clinical symptomatology especially impaired behavioral and social interactions [21].

Moreover, the current study revealed significantly lower morning basal cortisol, higher ACTH and lower cortisol response after ACTH stimulation as the autistic severity increased. In addition, CARS score correlated positively with ACTH and negatively with each of basal and stimulated cortisol. This means that the extent of the HPA axis dysfunction was closely linked to autistic severity which was confirmed by another study [30].

In the current study, all hormonal parameters did not differ significantly according to the IQ of autistic subjects. Similarly, Marinovic-Curin etal, 2003 [21] and Tordjman etal 1997 [30] concluded that IQ itself did not appear to influence the hormonal levels significantly bearing in mind that they did not confirm their results by performing an ACTH stimulation test. To the best of our knowledge, we could not trace data in literature to explain the mechanism by which low plasma cortisol could affect cognitive functions in autistic children. On the other hand, another study [39] concluded that, generally speaking, increased cortisol levels due to stress-induced HPA axis over activity have been associated with cognitive impairment as stress-induced HPA axis overactivity and increased cortisol levels may cause hippocampal damage and, subsequently, cognitive decline. Furthermore, patients with Cushing's syndrome more often present with cognitive impairment [40]. Finally, one of the side-effects of treatment with synthetic corticosteroids is cognitive deterioration [41]

Also, the current study revealed that hormonal profile did not differ significantly between autistic children with and without EEG abnormalities which was also supported by Marinovic-Curin and associates [21].

In addition, the current study demonstrated that hormonal profile did not significantly differ between autistic patients with and without self injurious behavior which was also confirmed by another study [21]. Inspite of the previous fact, there is evidence of an association between low HPA axis activity and antisocial behavior since cortisol, which is the final product of the HPA axis, is an important factor for proper emotional and social development and functioning [42,43].

## Conclusions

In conclusion, the observed hormonal changes may be due to a dysfunction in the HPA axis in autistic individuals. Further studies are warranted regarding the role of HPA axis dysfunction in the pathogenesis of autism.

## Competing interests

The authors declare that they have no competing interests.

## Authors' contributions

RTH conceived the study, participated in its design and coordination, drafted the manuscript, helped in collection of demographical and clinical data of the children and gave final approval of the version to be published. DHH conceived the study; and participated in its design and coordination and collected demographical and clinical data of the children. MAI carried out the laboratory studies and performed the statistical analysis. All authors read and approved the final manuscript

## Authors' information

- RTH: Assistant Professor of Pediatrics and Pediatric Endocrinology, Ain Shams University, Cairo, Egypt.

- DHH: Assistant Professor of Psychiatry, Ain Shams University, Cairo, Egypt.

- MAI: Assistant Professor of Clinical Pathology, Ain Shams University, Cairo, Egypt.

## References

[B1] Lainhart JE Developmental abnormalities in autism Lancet 1997 349 373 374 10.1016/S0140-6736(97)80005-0 9033460

[B2] Xue M Chaaban J Zimmerman-Bier B Wagner GC Autism spectrum disorders: concurrent clinical disorders J Child Neurol 2008 23 6 13 18056691 10.1177/0883073807307102

[B3] Dawson G The search for autism's roots Nature 2001 41 882 884 10.1038/35082228 11418823

[B4] Stokstad E New hints into the biological basis of autism Science 2001 294 34 37 10.1126/science.294.5540.34 11588233

[B5] Wolft S The history of autism Eur Child Adolesc Psychiatry 2004 13 201 208 10.1016/S1056-4993(03)00095-6 15365889

[B6] Shao Y Cuccaro ML Hauser ER Raiford KL Menold M Wolpert CM Fine mapping of autistic disorder to chromosome15q11-q13 by use of phenotypic subtypes Am J Hum Genet 2003 72 539 548 10.1086/367846 12567325 PMC1180230

[B7] Veenstra-Van Der Weele J Cook EH Molecular genetics of autism spectrum disorder Mol Psychiatry 2004 7 73 78 10.1038/sj.mp.4001505 15197396

[B8] Wassink TH Brzustowicz LM Bartlett CW Szatmari P The search of autism disease genes Ment Retard Dev Disabil Res Rev 2004 10 272 283 10.1002/mrdd.20041 15666342

[B9] Jansen LM Gispen-De Wied CC Van Der Gaag RJ Van Engeland F Differentiation between autism and multiple complex developmental disorder in response to psychosocial stress Neuropsychopharmachology 2003 28 582 590 10.1038/sj.npp.1300046 12629541

[B10] Strous RD Golubchik P Maayan R Mozes T Tuati-Werner D Weizman A Lowered DHEA-S plasma levels in adult individuals with autistic disorder Eur Neuropsychopharmacol 2005 15 305 309 10.1016/j.euroneuro.2004.12.004 15820420

[B11] Trottier G Srivastava L Walker CD Etiology of infantile autism: a review of recent advances in genetic and neurobiological research J Psychiatry Neurosci 1999 24 103 115 10212552 PMC1188990

[B12] Aylward EH Minshev NJ Goldstein G Honeycutt NA Augustine AM Yates KO MRI volumes of amygdala and hippocampus in not mentally retarded autistic adolescents and adults Neurology 1999 53 2145 2150 10599796 10.1212/wnl.53.9.2145

[B13] Palmen SJ Van England H Hof PR Shmitz C Neuropathological findings in autism Brain 2004 127 2572 2583 10.1093/brain/awh287 15329353

[B14] Rojas DC Smith JA Benkers TL Camou SL Reite ML Rogers SJ Hippocampus and amygdala volume in parents of children with autistic disorder Am J Psychiatr 2004 161 2038 2044 10.1176/appi.ajp.161.11.2038 15514404

[B15] Bailey A LeCouteur A Smonoff E Yuzda E Autism as a strongly genetic disorder: Evidence from a British twin study Psychological Medicine 1995 25 63 78 10.1017/S0033291700028099 7792363

[B16] Cohly HH Panja A Immunological findings in autism Int Rev Neurobiol 2005 71 317 341 10.1016/S0074-7742(05)71013-8 16512356

[B17] Hrdlicka M Dudlova I Beranova I Lisy J Belsan T Neuwirth J Subtypes of autism by cluster analysis based on srtuctural MRI data Eur Child Adolesc Psychiatry 2005 14 138 144 10.1007/s00787-005-0453-z 15959659

[B18] Jacobson L Hypothalamic-pituitary-adrenocortical axis regulation Endocrinol Metab Clin North Am 2005 34 271 292 10.1016/j.ecl.2005.01.003 15850842

[B19] Vedhara K Hyde J Gilchrist ID Tytherleigh M Plummer S Acute stress, memory, attention and cortisol Psychoneuroendocrinology 2000 25 535 549 10.1016/S0306-4530(00)00008-1 10840167

[B20] Corbett BA Mendosa S Abdullah M Wegelin JA Levine S Cortisol circadian rhythms and response to stress in children with autism Psychoneuroendochrinology 2006 31 59 68 10.1016/j.psyneuen.2005.05.011 16005570

[B21] Marinovic-Curin J Terzic J Bujas-Petkovic Z Zekan LJ Marinovic-Terzic I Marasovic-Susnjara I Lower cortisol and higher ACTH levels in individuals with autism J Autism Dev Disord 2003 33 443 448 10.1023/A:1025019030121 12959423

[B22] Richdale AL Prior MR Urinary cortisol circadian rhythm in a group of high functioning children with autism J Autism Dev Disord 1992 22 433 447 10.1007/BF01048245 1400105

[B23] American Psychiatric Association Diagnostic and Statistical Manual of Mental Disorders 1994 4 Washington DC: American Psychiatric Association

[B24] Tanner JM Whitehouse RH Clinical longitudinal standards for height, weight, height velocity, weight velocity, and stages of puberty Arch Dis Child 1976 51 170 179 10.1136/adc.51.3.170 952550 PMC1545912

[B25] Schopler E Reichler RJ Renner BR The Childhood Autism Rating Scale (CARS), for Diagnostic Screening and Classification in Autism 1986 New York: Irvington

[B26] Wechsler D Wechsler intelligence scale for children 1991 3 NewYork: Psychological Corporation. Harcourt Brace and Company

[B27] Babson AL The Immulite 2000. Automated immunoassay system J Clin Immunoassy 1991 14 83 88

[B28] Yoshimura K Naiki Y Horikawa R Tanaka T Three Patients with Autism and Central Precocious Puberty Clinical Pediatric Endocrinology 2005 14 55 57 10.1297/cpe.14.S24_55

[B29] Adolphs R Tranel D Damasio H Damasio A Impaired recognition of emotion in facial expressions following bilateral damage to the human amygdala Nature 1994 372 669 672 10.1038/372669a0 7990957

[B30] Tordjman S Anderson GM McBride PA Hertzing ME SnowM E Hall LM Plasma beta-endorphin, adrenocorticotropin hormone, and cortisol in autism Journal of Child Psychology and Psychiatry 1997 38 705 715 10.1111/j.1469-7610.1997.tb01697.x 9315980

[B31] Herman BH Arthur-Smith A Hammock MK Josephs S Ontogeny of beta-endorphin and cortisol in plasma of children and adolescents J Clin Endocrinol Metab 1988 67 186 189 10.1210/jcem-67-1-186 2967851

[B32] Tani P Lindberg N Matto V Appelberg B Nieminen-Von Wendt T Von Wendt L Higher plasma ACTH levels in adults with Asperger syndrome J Psychosom Res 2005 58 533 536 10.1016/j.jpsychores.2004.12.004 16125520

[B33] Van Heringen K Van De Viele Verstraete A Cortisol in violent behavior: Association with personality and monoaminergic activity Journal of Affective Disorders 2000 60 181 189 10.1016/S0165-0327(99)00180-9 11074106

[B34] Yehuda R Bierer LM Schmeidler J Aferiat DH Breslau I Dolan S Low cortisol and risk for PTSD in adult offspring of holocaust survivors Am J Psychiatr 2000 157 1252 1259 10.1176/appi.ajp.157.8.1252 10910787

[B35] Gispen- De Wied CC Jansen LM Duyx JH Thijssen JH Van Engeland H Pituitary-adrenal function in adolescent psychiatric patients: Impact of depressive symptoms Journal of Affective Disorders 2000 59 71 76 10.1016/S0165-0327(99)00116-0 10814774

[B36] Bouvard MP Leboyer M Launay JM Recasens C Plumet M Waller-Perotte D Low-dose naltrexone effects on plasma chemistries and clinical symptoms in autism: A double blind, placebo-controled study Psychiatr Res 1995 58 191 201 10.1016/0165-1781(95)02601-R 8570775

[B37] Leboyer M Bouvard MP Launay JM Tabateau D Waller D Dugas M Brief report: A double-blind study of naltrexone in infantile autism J Autism Dev Disord 1992 22 309 319 10.1007/BF01058158 1345670

[B38] Leboyer M Bouvard MP Recasens C Philippe A Guilloud- Bastile MF Bondoux D Difference between plasma N- and C-terminally directed endorphin immunoreactivity in infantile autism Am J Psychiatr 1994 151 1797 1810 7977888 10.1176/ajp.151.12.1797

[B39] Kalmijn S Launer LJ Stolk RP De Jong FH Pols HAP Hofman A A Prospective Study on Cortisol, Dehydroepiandrosterone Sulfate, and Cognitive Function in the Elderly J Clin Endocrinol Metab 1998 3 10 3487 3492 10.1210/jc.83.10.3487 9768651

[B40] Mauri M Sinforiani E Bono G Vignati F Berselli ME Attanasio R Memory impairment in Cushing's disease Acta Neurol Scand 1993 87: 52 55 8424312 10.1111/j.1600-0404.1993.tb04075.x

[B41] Wolkowitz OM Prospective controlled studies of the behavioral and biological effects of exogenous corticosteroids Psychoneuroendocrinology 1994 19 233 10.1016/0306-4530(94)90064-7 7515507

[B42] Sapolsky RM Why stress is bad for your brain Science 1996 273 749 750 10.1126/science.273.5276.749 8701325

[B43] Raine A Biosocial studies of antisocial and violent behavior in children and adults: a review J Abnorm Child Psychol 2002 30 311 326 10.1023/A:1015754122318 12108763

